# Fatigue: assess and address it

**DOI:** 10.1186/s41687-026-01188-w

**Published:** 2026-08-25

**Authors:** Tanja A. Kuut, Hans Knoop, Hedy A. van Oers, Lotte Haverman

**Affiliations:** 1 Department of Medical Psychology, Amsterdam UMC Location University of Amsterdam, Amsterdam, Netherlands; 2 Amsterdam Public Health, Mental Health, Amsterdam, Netherlands; 3 Emma Children’s Hospital, Child and Adolescent Psychiatry & Psychosocial Care, Amsterdam UMC Location University of Amsterdam, Amsterdam, Netherlands

**Keywords:** Fatigue, patient-reported outcome measures, Transdiagnostic, Non-pharmacological treatments for fatigue

## Abstract

Severe fatigue is highly prevalent in patients across different medical conditions and can only be partially explained by disease-specific factors. Also, fatigue is often seen by patients as one of the most disabling symptoms. Despite its substantial impact, fatigue is often not systematically discussed in clinical practice. Effective generic treatments exist for patients with severe fatigue. When fatigue is not assessed, patients may be withhold the opportunity to get advice on how to manage fatigue or receive effective interventions to achieve meaningful improvement. Therefore, designated health care professionals involved in patient care should be able to recognize and address fatigue as a key patient-reported outcome.

**To the editor**

Fatigue is a debilitating symptom experienced by patients across a wide range of medical conditions [1]. Therefore, it is essential that it receives adequate attention during clinical encounters and throughout treatment. However, in our experience, this is not yet consistently the case in routine hospital care. Fatigue is often not systematically discussed in clinical practice, and sometimes even neglected. As the Amsterdam UMC patient-reported outcome measure (PROM) Expertise Centre, we have been working on the implementation of PROMs in routine clinical practice for over 15 years and implemented PROMs for more than 160 healthcare teams in collaboration with patients and health care professionals [2]. The implementation of PROMs in routine clinical care follows a structured, stepwise process. It begins with clinicians completing an intake form describing the objectives of the implementation, the patient population, and any preferred patient-reported outcomes (PROs) and PROMs. The most relevant PROs are identified through a review of the literature, discussion with the clinical team and/or focus groups with patients. Based on the prioritized PROs, the most appropriate PROMs are then selected [2]. We have observed during this implementation process as well as identified in literature studies that fatigue is often regarded by health care professionals as a less relevant symptom to assess, while patients find it an important symptom to assess. While clinicians are generally aware that fatigue is a common issue, they may often feel that there is little they can do to treat or to help patients effectively manage it. This is unfortunate since effective treatments exist for patients with severe fatigue and psycho-education can help patients to better manage the symptom. In this letter, we combine our experience with the expertise of clinicians and researchers of a tertiary treatment centre for chronic fatigue at the Amsterdam UMC. Bringing together our expertise we will support our observations with the literature and advocate for a stronger focus on fatigue by health care professionals involved in patient care. Our aim is to provide clinicians practical guidance to measure fatigue, in order to address fatigue as part of truly patient-centred care.

Fatigue is a common symptom in the general population. Compared with the general population, a substantially higher proportion of patients with a long-term medical condition is severely and chronically fatigued with prevalence rates ranging from 25 up to 70% [1]. Multi-morbidity increases the likelihood of having severe and chronic fatigue [1]. Severe fatigue is often reported by patients as their most burdensome symptom. Further, severe fatigue is closely associated with functional limitations and health related disability [3]. Severe fatigue often has a chronic course [1]. Severe fatigue is associated with higher health care use and societal costs. A panel of patients prioritised fatigue as the most important topic that should be addressed to improve clinical care [4]. These findings warrant paying attention to fatigue by healthcare professionals.

Chronic fatigue in long term medical conditions often can only be partly explained by disease-specific factors [1]. It often has a weak association with somatic markers of disease and disease activity [3]. As objective somatic markers of fatigue are unknown, we have to rely on patients’ experiences for its assessment. This highlights the importance of measuring fatigue using PROMs completed by patients. PROMs capturing patients’ own perspectives on how they feel, function and live with their condition by generic, validated instruments offer a valuable complement to clinical assessments and lab results, especially when assessing fatigue. Originally developed for research, PROMs are now widely used in clinical care to monitor symptoms, guide treatment, and support shared decision-making. Systematic reviews confirm that PROMs positively impact clinical care processes and outcomes, where they improve quality of life, satisfaction, patient-clinician communication and symptom management with moderate-certainty evidence [5]. A cluster-randomized controlled trial demonstrated that PRO monitoring significantly improved symptoms and physical function [6]. Fatigue showed consistent improvement across all time points in the intervention group, suggesting that structured symptom monitoring enables earlier recognition and better management of this burdensome symptom [4].

Despite this, several studies suggest that while professionals acknowledge the need to address fatigue and educate patients on how one can manage it, this is not mirrored in patient experiences [7]. One study indicated a discrepancy in perception as a higher percentage of clinicians reported discussing complaints like fatigue compared to patients, suggesting that attention to fatigue is perceived differently by clinicians and patients [8]. One of the most pronounced discrepancies between clinician and patient symptom ratings has been observed in the assessment of fatigue, with clinicians consistently underestimating its severity and impact compared to patients’ self-reports [9]. Also, fatigue is often not included in the set of PROMs advised for use in clinical care and research in clinical care. An analysis from 2021 examined the extent to which PROs overlap across the 39 ICHOM Standard Sets across different health conditions. Fatigue appeared only in 10 of the 39 sets (26%) [10]. In the additional 11 ICHOM sets recently published three included fatigue (27%). This suggests that fatigue is not yet universally recognized as a core outcome. The finding that physicians often feel frustrated, ill-equipped, and time-pressured when managing patients with fatigue might be a reason for physicians not to assess or address fatigue [11].

Fatigue is closely related to transdiagnostic biopsychosocial factors including behavioural factors like physical activity, sleep-related factors, depression and cognitive factors like self-efficacy with respect to fatigue [1]. Accordingly, most effective interventions for fatigue are generic instead of disease-specific [12]. However, to ensure that patients are referred to such interventions, fatigue must be identified, and the tendency among clinicians to avoid discussing fatigue is therefore worrying. Not assessing fatigue can mean withholding the patient from the possibility to improve. A high fatigue score on a PROM can prompt the treating physician to discuss its management and potential treatment options. A first step is providing the patient with specific education about fatigue and the difference between normal and severe persistent fatigue [13]. Further, advice on general strategies like sleep hygiene and encouraging physical activity can be provided [13]. When this turns out not being sufficient, the patient can be referred for non-pharmacological treatments. Several non-pharmacological treatments for fatigue have been shown effective, including cognitive behavioural therapy targeting fatigue, mindfulness-based interventions and physical activity programs [12, 13].

Fatigue should be routinely addressed and discussed during clinical encounters, with psychoeducation provided as a standard component of care. When indicated, referral to specialized support should be offered. To support this aspects of care, we recommend that and fatigue should be more consistently included in PRO sets and PROMs assessing fatigue should be integrated into routine care [13].

We realize that this comes with various challenges. First, many barriers hindering the implementation of PROMs in clinical care have been described, such as limited time of health care professionals, limited resources, uncertainty regarding the clinical value of PROMs and challenges related to patient engagement [14]. Further, many health care professional are not trained in communication about fatigue and how to refer to a non-pharmacological intervention. In addition, greater emphasis is needed on further improvement of transdiagnostic interventions for severe fatigue, alongside efforts to improve their accessibility in routine practice [15].

In conclusion, we would like to advocate that designated clinicians should be able to recognize, address, and systematically assess fatigue as a key patient-reported outcome. Routinely addressing fatigue through discussing the outcomes of PROMs, targeted psychoeducation, advice how to cope with fatigue and timely referrals is essential to improve patient–clinician communication, reduce symptom burden, and ultimately enhance patients’ quality of life and functioning.

## Data Availability

No datasets were generated or analysed during the current study.
